# Supplementary material for: Comparing the Toxicological Responses of Pulmonary Air–Liquid Interface Models upon Exposure to Differentially Treated Carbon Fibers
Source: Int J Mol Sci. 2023 Jan 18;24(3):1927. doi: 10.3390/ijms24031927 (PMC9915385; doi:10.3390/ijms24031927)
Supplement: Supplementary file 1 [file ijms-24-01927-s001.zip › ijms-2118400-supplementary.pdf]

Supplementary materials

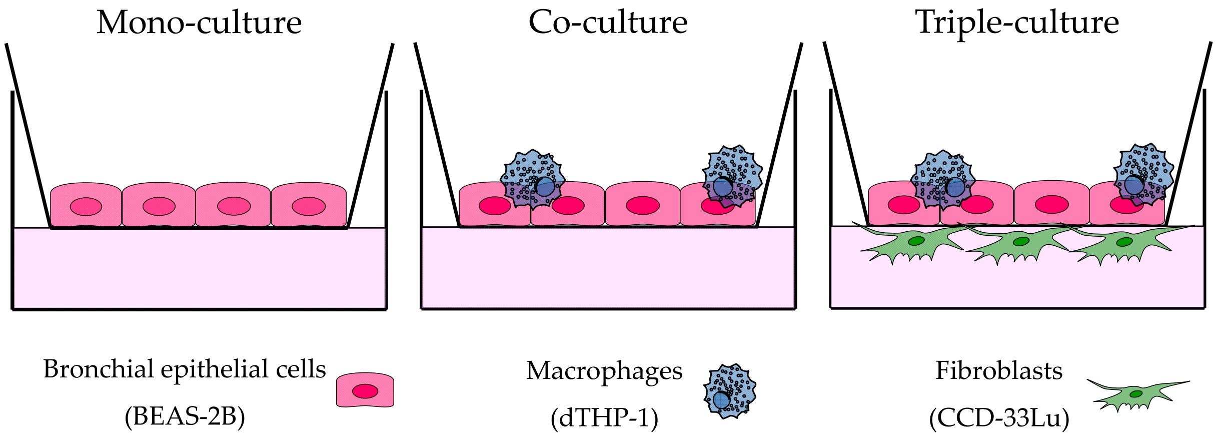

Figure S1. Schematic representation of the three cell culture models used for CF exposure experiments. The mono-cultures were composed of the bronchial epithelial cell line BEAS-2B grown in the apical compartment of a Transwell® insert. For a co-culture model, differentiated macrophage-like cells (dTHP-1) were added into the apical compartment. For a triple-culture, fibroblasts were attached to the basolateral side of the Transwell® membranes prior to the seeding of BEAS-2B und dTHP-1 cells.

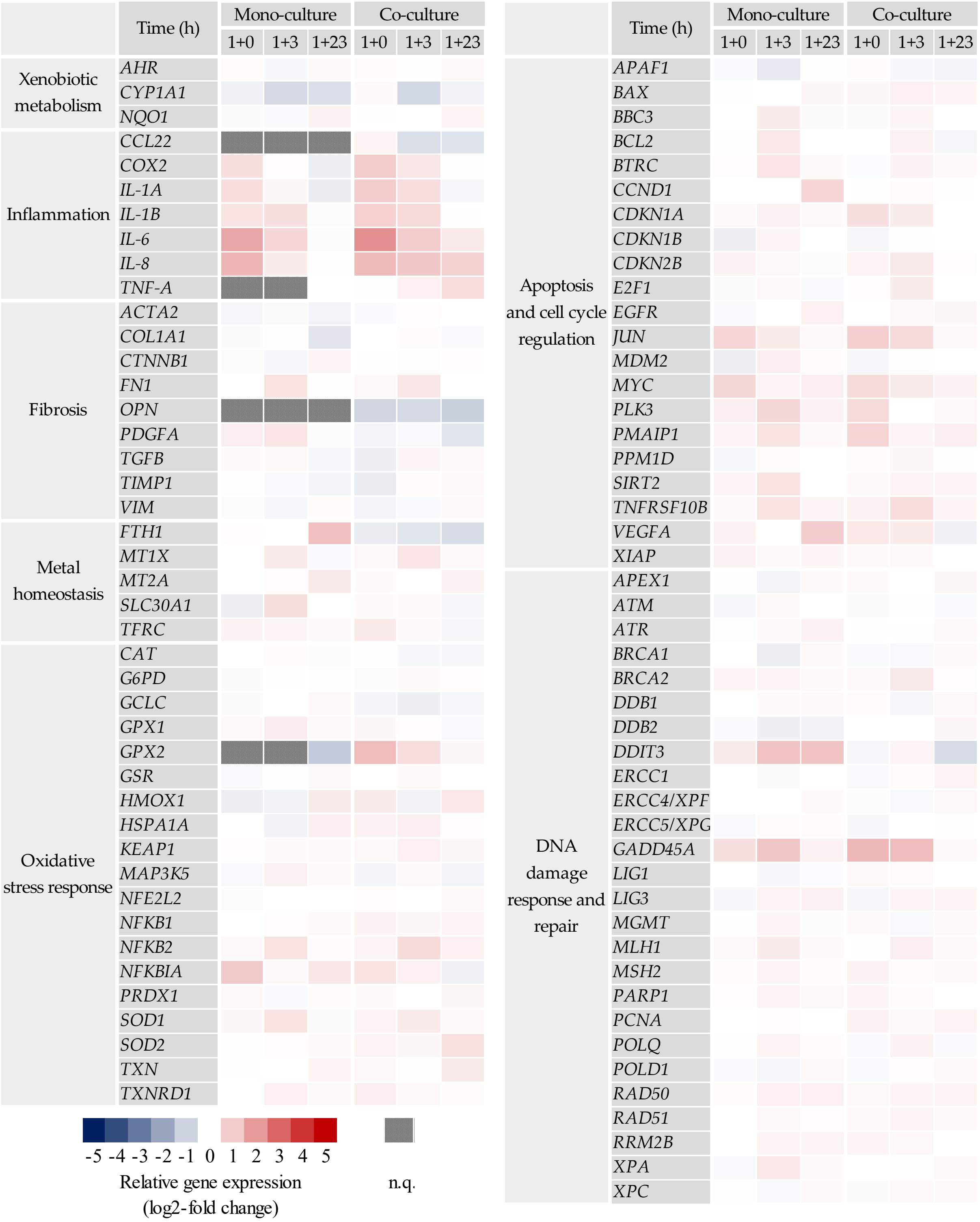

Figure S2. Full gene expression profiles of BEAS-2B mono- and BEAS-2B/dTHP-1 co-cultures after exposure to mechanically treated CF. Both cell cultures were exposed to 7.00± 1.25 µg/cm² CF for 1 h and post-incubated for 0, 3 or 23 h. The results are depicted as the log2-fold change of the relative gene expression. A red color represents the induction, a blue color the repression of a gene. The means of at least three independent experiments are displayed. n.q.: not quantifiable due to low expression levels.

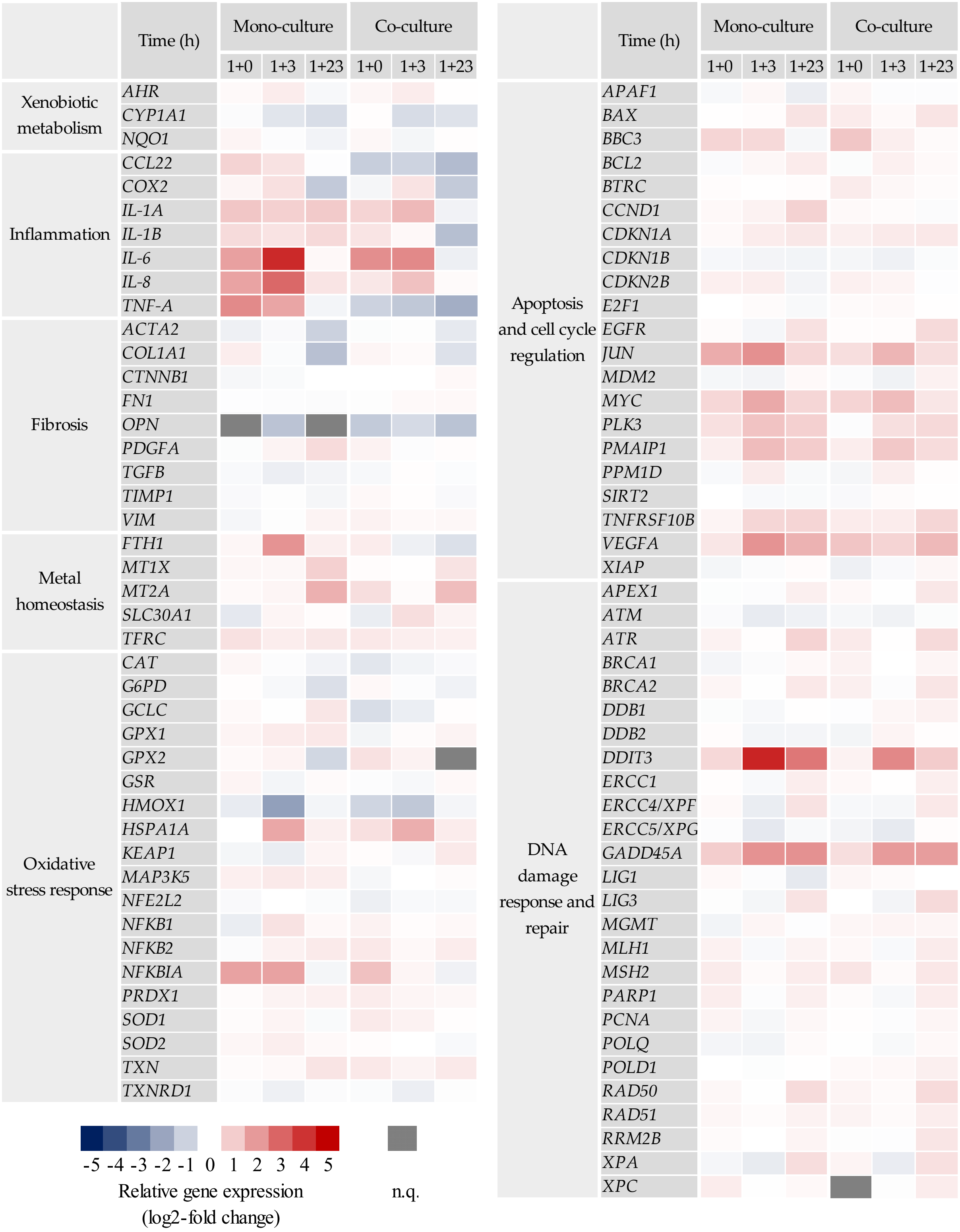

Figure S3. Full gene expression profiles of BEAS-2B mono- and BEAS-2B/dTHP-1 co-cultures after exposure to thermo-mechanically treated CF. Both cell cultures were exposed to 6.55 ± 1.96 µg/cm² CF for 1 h and post-incubated for 0, 3 or 23 h. The results are depicted as the log2-fold change of the relative gene expression. A red color represents the induction, a blue color the repression of a gene. The means of at least three independ-ent experiments are displayed. n.q.: not quantifiable due to low expression levels.

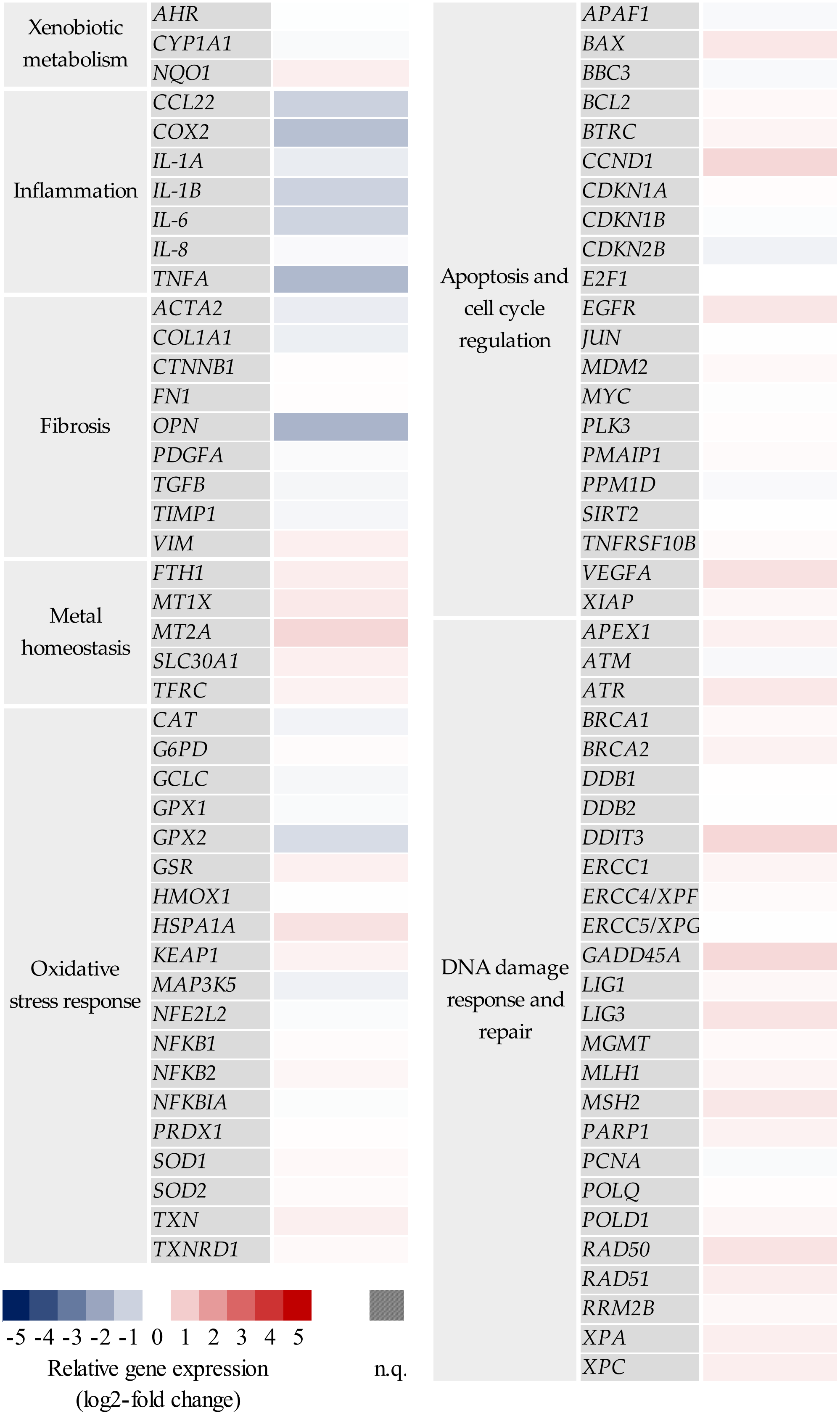

Figure S4. Full gene expression profiles of BEAS-2B/dTHP-1/CCD-33Lu triple-cultures after exposure to mechanically treated CF. Triple-cultures were exposed to 7.00± 1.25 µg/cm² CF for 1 h and post-incubated for 23 h. The results are depicted as the log2-fold change of the relative gene expression. A red color represents the induction, a blue color the repression of a gene. The means of at least three independent experiments are displayed. n.q.: not quantifiable due to low expression levels.

Table S1. Complete list of genes utilized in the HT RT-qPCR with their corresponding proteins.

Cluster	Gene	Corresponding protein
Xenobiotic metabolism	*AHR*	aryl hydrocarbon receptor
	CYP1A1	cytochrome P450, family 1, subfamily A, polypeptide 1 (CYP1A1)
	NQO1	NAD(P)H dehydrogenase, quinone 1 (NQO1)
Inflammation	*CCL22*	C-C motif chemokine 22 (CCL22)
	COX2	prostaglandin-endoperoxide synthase 2 (PTGS2)/ cyclooxygenase-2 (COX-2)
	IL-1A	interleukin-1α (IL-1α)
	IL-1B	interleukin-1β (IL-1β)
	IL-6	interleukin-6 (IL-6)
	IL-8	interleukin-8 (IL-8)
	TNF-A	tumor necrosis factor α (TNF-α)
Fibrosis	*ACTA2*	alpha smooth muscle actin (α-SMA)
	COL1A1	collagen, type I, alpha 1 (COL1A1)
	CTNNB1	β-catenin (CTNNB)
	FN1	fibronectin1 (FN1)
	OPN	osteopontin (OPN)
	PDGFA	platelet-derived growth factor subunit A (PDGF-A)
	TGFB	transforming growth factor β (TGF-β)
	TIMP1	tissue inhibitor of metalloproteinase (TIMP1)
	VIM	vimentin (VIM)
Metal homeostasis	*FTH1*	ferritin, heavy polypeptide 1 (FTH1)
	MT1X	metallothionein 1X (MT1X)
	MT2A	metallothionein 2A (MT2A)
	SLC30A1	solute carrier family 30 (zinc transporter), member 1 (ZnT1)
	TFRC	transferrin receptor (TFR)
Oxidative stress response	*CAT*	catalase (CAT)
	G6PD	glucose-6-phosphate dehydrogenase (G6PD)
	GCLC	glutamate-cysteine ligase, catalytic subunit (GCL)
	GPX1	glutathione peroxidase 1 (GPX1)
	GPX2	glutathione peroxidase 2 (GPX2)
	GSR	glutathione reductase (GSR)
	HMOX1	heme oxygenase (decycling) 1 (HO1)
	HSPA1A	heat shock 70kDa protein 1A (hsp70)
	KEAP1	kelch-like ECH-associated protein 1 (Keap1)
	MAP3K5	mitogen-activated protein kinase kinase kinase 5 (MAP3K5/ASK1)
	NFE2L2	nuclear factor, erythroid 2-like 2 (Nrf2)
	NFKB1	nuclear factor of kappa light polypeptide gene enhancer in B-cells 1 (p50/p105)
	NFKB2	nuclear factor of kappa light polypeptide gene enhancer in B-cells 2 (p49/p100)
	NFKBIA	nuclear factor of kappa light polypeptide gene enhancer in B-cells inhibitor,alpha (IKBA)
	PRDX1	peroxiredoxin 1 (Prx1)
	SOD1	superoxide dismutase 1, soluble (SOD1)
	SOD2	superoxide dismutase 2, mitochondrial (SOD2/MnSOD)

	TXN	thioredoxin (Txr)
	TXNRD1	thioredoxin reductase 1 (TxrR)
Apoptosis and cell cycle regulation	*APAF1*	apoptotic protease activating factor (APAF1)
	BAX	bcl2-associated x protein (bax)
	BBC3	p53 up-regulated modulator of apoptosis (PUMA)
	BCL2	b-cell cll/lymphoma 2 (bcl-2)
	BTRC	transducin repeat containing E3 ubiquitin protein ligase, beta (β-TrCP)
	CCND1	cyclin D1 (CCND1)
	CDKN1A	cyclin-dependent kinase inhibitor 1A (p21)
	CDKN1B	cyclin-dependent kinase inhibitor 1B (p27)
	CDKN2B	cyclin-dependent kinase inhibitor 2B (p15)
	E2F1	E2F transcription factor 1 (E2F1)
	EGFR	epidermal growth factor receptor (EGFR)
	JUN	jun proto-oncogene (c-jun)
	MDM2	Mouse double minute 2 hom. proto-onco-gene, E3 ubiquitin protein ligase (Mdm2)
	MYC	v-myc avian myelocytomatosis viral oncogene homolog (c-myc)
	PLK3	polo-like kinase 3 (PLK3)
	PMAIP1	phorbol-12-myristate-13-acetate-induced protein 1 (Noxa)
	PPM1D	protein phosphatase, mg2+/Mn2+ dependent, 1D (PPM1D)
	SIRT2	sirtuin 2 (SIRT2)
	TNFRSF10B	tumor necrosis factor receptor superfamily, member 10b (DR5)
	VEGFA	vascular endothelial growth factor A (VEGFA)
	XIAP	X-linked inhibitor of apoptosis (XIAP)
DNA damage response and repair	*APEX1*	apurinic-apyrimidinic endonuclease 1 (APEX1/Ref-1)
	ATM	ataxia telangiectasia mutated (ATM)
	ATR	ataxia telangiectasia and Rad3-related protein (ATR)
	BRCA1	breast cancer 1, early onset (BRCA1)
	BRCA2	breast cancer 2, early onset (BRCA2)
	DDB1	damage-specific DNA binding protein 1 (DDB1)
	DDB2	damage-specific DNA binding protein 2 (DDB2)
	DDIT3	growth arrest and DNA damage-inducible protein (GADD153)
	ERCC1	excision repair cross-complementation group 1 (ERCC1)
	ERCC4/XPF	excision repair cross-complementation group 4 (XPF)
	ERCC5/XPG	excision repair cross-complementation group 5 (XPG)
	GADD45A	growth arrest and DNA-damage-inducible, alpha (GADD45A)
	LIG1	ligase I, DNA, ATP-dependent (LIG1)
	LIG3	ligase III, DNA, ATP-dependent (LIG3)
	MGMT	O-6-methylguanine-DNA methyltransferase (MGMT)
	MLH1	mutL homolog 1 (MLH1)
	MSH2	mutS homolog 2 (MSH2)
	PARP1	Poly (ADP-ribose) polymerase 1 (PARP1)
	PCNA	proliferating cell nuclear antigen (PCNA)
	POLB	polymerase (DNA directed), beta (POLB)

POLQ	
RAD50	RAD50 homolog (S. cerevisiae) (RAD50)
RAD51	RAD51 recombinase (RAD51)
RRM2B	ribonucleotide reductase M2 B (TP53 inducible) (p53R2)
XPA	xeroderma pigmentosum, complementation group A (XPA)
XPC	xeroderma pigmentosum, complementation group C (XPC)

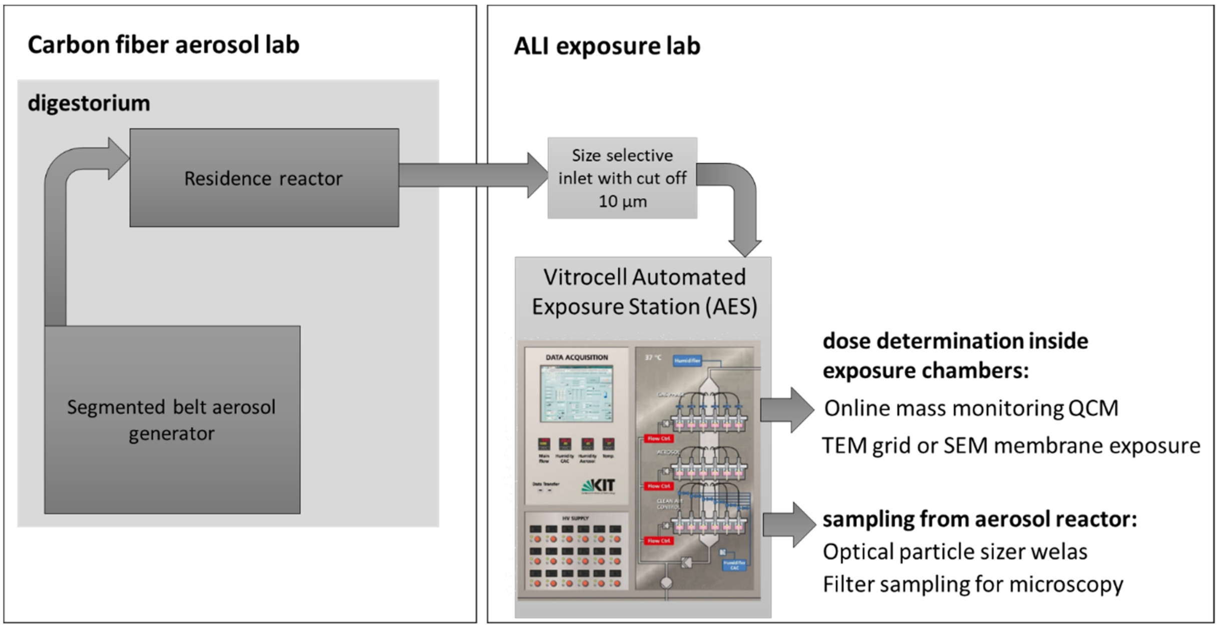

Figure S5. Schematic representation of the setup for ALI exposure studies. In the carbon fibre aerosol lab the CF were dispersed by the segmented belt aerosol generator and after passing the residence reactor the CF aerosol is brought to the air-liquid interface (ALI) exposure lab where the Vitrocell AES is located. QCM = quartz crystal microbalance, SEM = scanning electron microscopy, TEM = transmission electron microscopy.

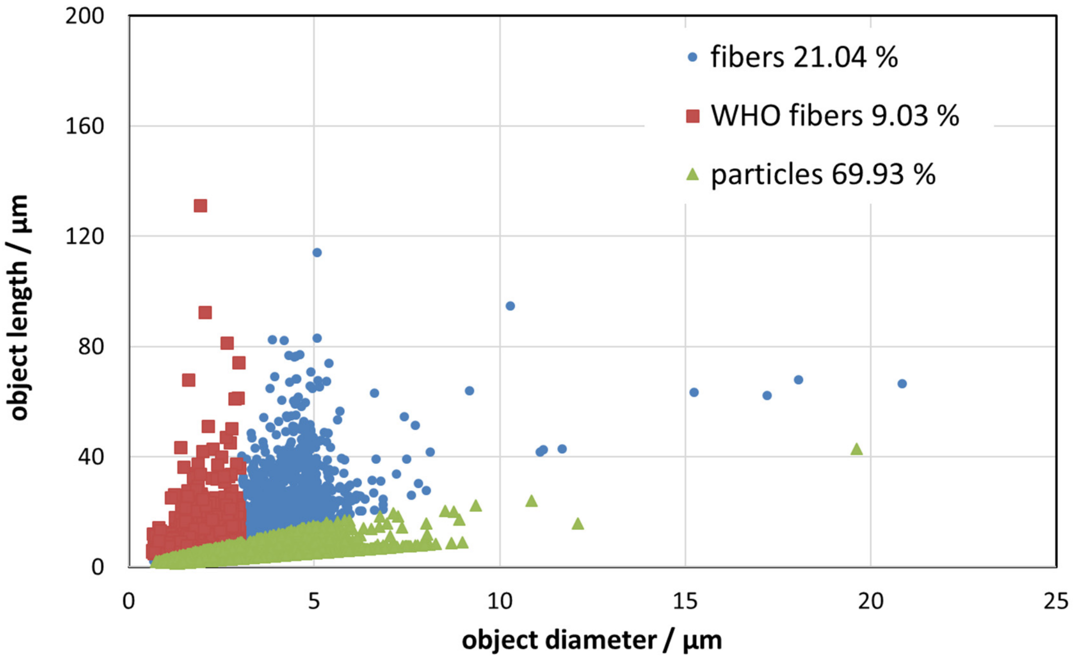

Figure S6. Representative single object analysis of a CF sample subjected to thermal-mechanical treatment. The color code indicates the classification of an object as particle (green triangle). fiber (blue dot) and WHO fiber (red square) dependent on the geometric properties length and diameter.
